# Combining glycine with thymoquinone offers a promising strategy for diabetes treatment

**DOI:** 10.1038/s41598-026-52735-w

**Published:** 2026-05-19

**Authors:** Nourhan N. Bash, Entsar A. Saad, Ibrahim H. El-Sayed, Kholoud H. Radwan

**Affiliations:** 1 Faculty of Science, Chemistry Department, Damietta University, New-Damietta, Damietta 34517 Egypt; 2 Faculty of Science, Chemistry Department, Kafr El-Sheikh University, Kafr El-Sheikh, Egypt; 3 Faculty of Physical Therapy, Department of Basic Science, Horus University-Egypt, New Damietta, 34518 Egypt

**Keywords:** β cells, Diabetes, Inflammation, Insulin resistance, Oxidative stress, Pancreas, Biochemistry, Diseases, Drug discovery, Endocrinology, Physiology

## Abstract

Diabetes is increasing globally, and current treatments are frequently associated with high costs and harmful side effects. Researchers are exploring safer, natural alternatives as thymoquinone and glycine. Glycine, the simplest proteinogenic amino acid, and thymoquinone, the active compound in *Nigella sativa*, have shown promising hypoglycemic, antioxidant, and anti-inflammatory properties. A combination of glycine and thymoquinone for treating diabetes was not investigated before. This work studies the biochemical and histopathological effects of thymoquinone and glycine administered in combination in a diabetic rat model. Diabetes mellitus was induced by streptozotocin (STZ). Five rat groups were used: control healthy, diabetes, diabetic rats treated with glycine (G) or thymoquinone (Q), or the combination (Q + G) groups. Rat body weight changes, water and food intake, fasting blood glucose (FBG), hemoglobin (Hb), glycated Hb (HbA1C), insulin, HOMA-IR, HOMA %B and HOMA %S, QUICKI, albumin, amylase, creatine kinase, TNF-α, IL-10, glucose-6-phosphatase, fructose 1,6-bisphosphatase, glucose-6-phosphat dehydrogenase, oxidative stress portrait, and pancreas histopathology were investigated. Treatment with glycine or thymoquinone or both significantly decreased FBG, oxidative stress, and inflammation and improved metabolic dysfunction compared to untreated diabetic group. It also, revealed cytoprotection to pancreas tissue and reduced inflammatory infiltration. Effects of thymoquinone and glycine treatments were appreciated, however effects of thymoquinone combined with glycine were much better. In conclusion, thymoquinone and glycine combination, can effectively reduce both blood sugar and diabetic-associated complications through their synergistic, strong antioxidant and anti-inflammatory properties, which support the body’s natural cytoprotective ability.

**Supplementary Information:**

The online version contains supplementary material available at 10.1038/s41598-026-52735-w.

## Introduction

Insulin dependent diabetes mellitus (IDDM) is a chronic autoimmune disorder in which the body’s immune system gradually destroys the insulin-producing β-cells in the pancreas. As a result, individuals with IDDM lose the ability to produce insulin naturally and must depend on lifelong insulin therapy to regulate their blood glucose levels^1^. IDDM is a chronic condition that requires regular monitoring and support for body and mind. Although insulin delivery systems and glucose monitors have become more advanced, many people still find it difficult to maintain stable blood sugar levels. Daily management involves adjusting insulin doses, planning meals, and adapting exercise routines often without clear guidance. The expense of treatment and the worry about long-term complications add financial pressure and emotional strain, which can impair quality of life. To tackle these challenges effectively, continuous patient education and mental health support, including strategies to prevent diabetes overload, must be part of standard care^2^. IDDM is one of the most common chronic diseases of children and adolescents, but it can also occur in adults and represents 5–10% of all DM cases. In 2021, it was estimated that around 8.4 million people worldwide were living with IDDM, with 500,000 new cases reported that year. By 2040, the global number of individuals living with IDDM is expected to rise to between 13.5 and 17.4 million^3^. The disease mechanism of IDDM is more than just insulin deficiency, including complex metabolic disturbances including dysregulated glucose homeostasis, oxidative stress, chronic inflammation, and impair cellular function^4^. These complex complications demand treatment strategies designed to target not only glycemic control but also the underlying pathological mechanisms contributing to disease progression and diabetic complications^5^.

Medicinal plants in the form of extracts and their active constituents have been widely investigated for their biological activities and potential use in various therapeutic purposes. Researchers use both traditional knowledge and modern scientific methods to identify and validate these plant-derived compounds for use in medicines, intending to discover new treatments for various health conditions^6–12^. Health Organization (WHO) advocates for plant-based therapies as sustainable alternatives to conventional oral hypoglycemic drugs, which, despite their efficacy, pose safety concerns. Medicinal plants, rich in antioxidants and bioactive compounds, provide a richer and more varied molecular profile than synthetic drugs, making them highly promising for managing diabetes. However, clinical trials are essential to confirm their long-term safety and effectiveness^13^. There’s a growing interest in using natural bioactive compounds alongside traditional therapies to better manage diabetes. Among these, thymoquinone, main active ingredient of *Nigella sativa* seeds, has emerged as a compound with real therapeutic potential^14^. Recent studies have demonstrated that thymoquinone reduce diabetes-induced hepatic damage in rats via regulation of oxidative/nitrosative stress, apoptosis, and inflammatory cascades^15^. The compound has shown remarkable efficacy in reducing oxidative stress, improving metabolic parameters, lipid profile enhancement and improving glycemic control in diabetic rats, while also improving insulin secretion and hepatic glycogen storage in streptozotocin-induced diabetic models^15–17^. Thymoquinone helps fight diabetes in more than one way, working through several biological pathways. thymoquinone has been shown to improve glucose tolerance, reduce oxidative stress, reduce hepatic gluconeogenesis, normalize blood sugar and lipid imbalance, enhancing total antioxidant capacity (TAC) and stimulate insulin secretion, suggesting both protective and regenerative properties^13,17^. In diabetic rats, thymoquinone significantly reduces liver damage markers (AST, ALT, ALP), lipid peroxidation (MDA), and inflammatory cytokines (IL-1β, TNF-α, IL-6) while restoring antioxidant enzyme activity (CAT, GSH, T-SOD). Histological analysis confirmed that thymoquinone preserves liver structure and reduces collagen fiber buildup, minimizing diabetic complications. Furthermore, molecular docking analysis showed that thymoquinone binds to inflammatory and apoptotic proteins, inhibiting their activity^15,18^. Thymoquinone activates imidazoline receptors, leading to enhanced glucagon-like peptide-1 (GLP-1) secretion and improved glycemic control in diabetic rats. Blocking these receptors reduces thymoquinone’s effects, confirming their role in GLP-1 regulation. The findings suggest that thymoquinone may serve as a potential diabetes treatment by influencing incretin hormone pathways^19^.

Glycine is the simplest and major amino acid in humans. It is mainly generated in the liver and kidney and is used to produce collagen, creatine, glucose and purine. It is also involved in immune function, anti-inflammatory processes and anti-oxidation reactions. It has been noticed for its potential therapeutic benefits in diabetes management. In addition to its primary function in protein synthesis, glycine exhibits significant metabolic and protective functions. Researchers found that glycine supplementation can improve diabetes, obesity, hyperlipidemia, heart toxicity, and hypertension by enhancing glucose metabolism, insulin sensitivity and anti-inflammatory processes^20,21^. GlyNAC supplementation (a combination of glycine and N-acetylcysteine) improves mitochondrial function and insulin resistance in patients with type 2 diabetes. Researchers found that diabetic patients had impaired mitochondrial fatty-acid oxidation (MFO), increased mitochondrial glucose oxidation (MGO), and elevated insulin resistance (IR). After 14 days of GlyNAC supplementation, participants showed a 30% improvement in MFO, a 47% reduction in MGO, and a 22% decrease in IR, along with lower free-fatty acid (FFA) levels. These findings suggest that GlyNAC may help restore mitochondrial health and improve metabolic function in diabetes^22^. Researchers found that higher branched-chain amino acids (BCAA) levels are linked to increased diabetes risk, while higher Gly levels correlate with improved insulin sensitivity and β-cell function. The findings suggest that low glycine and elevated BCAA may serve as a biomarker for identifying risk for β cell failure^23^. Glycine supplementation significantly restores regular biorhythmic contraction and relaxation in pancreatic microcirculation, improves blood distribution patterns, and reverses endothelial oscillation impairment in IDDM mice. It also reduces inflammatory cytokines and protects against microvascular damage. This shows glycine may help prevent small blood vessel damage caused by diabetes, improving blood circulation and reducing complications^24^. Glycine is an amino acid that helps regulate the immune system in IDDM. When taken regularly, it can reduce inflammatory substances in the body and increase the production of a specific protein (interferon-gamma) that helps protect tissues from damage caused by chronic inflammation. Glycine works by affecting the activity of a key molecule in immune cells called NF-κB, making it a potential dietary supplement to support immunity and reduce inflammation^24,25^.

Although both thymoquinone and glycine show good effects on their own for IDDM in different ways, thymoquinone fights inflammation and protects insulin-producing cells, while glycine improves blood flow, enhances insulin sensitivity and regulates the immune system. Combining them could be even more effective, but more studies are needed. Moreover, while insulin therapy remains the gold standard for IDDM treatment, there is a great need for new therapeutic strategies designed to safely and effectively change how the disease develops over time during its sequential stages^26^.

Therefore, this study aims to evaluate and compare the therapeutic effects of thymoquinone and glycine, both individually and in combination, against streptozotocin (STZ)-induced diabetes mellitus in rats on both biochemical and histopathological levels. Therefore, this study was intended as the first one to study the effect of thymoquinone and glycine together against STZ-induced diabetes in rats. We believe that giving thymoquinone and glycine together might work better than using each one alone.

## Materials and methods

### Chemicals and kits

STZ and thymoquinone were purchased from Cronelle Lab, Cairo, Egypt. Glycine (99% assay) was purchased from El Nasr Pharmaceutical Chemicals, Egypt. Other chemicals and reagents of the highest available pure grade were used. The hemoglobin kit was purchased from BIO-DIAGNOSTIC, Giza, Egypt. The FBG and HbA1C kits were purchased from HUMAN COMPANY, Germany. Rat Insulin, INS ELISA Kit was purchased from Cusabio, Wuhan, China. Creatinine kinase (CK)-T and CK-MB were purchased from SPIN REACT, Ctra, Santa Coloma, Spain. Tumor necrosis factor-alpha (TNF-α), interleukin-10 (IL-10), glucose-6-phosphate, glucose-6-phosphate dehydrogenase, and fructose 1,6 bis-phosphatase ELISA immunoassay kits were purchased from FINE BIOTECHNOLOGY, China. Liver homogenate malondialdehyde (MDA), glutathione reduced form (GSH), and total antioxidant capacity (TAC) were purchased from BIO-DIAGNOSTIC, Giza, Egypt.

### Experimental animals

Animals were 42 adult male Swiss albino rats (weighing 150–200 g, aged 6–8 weeks) obtained from Urology and Nephrology Centre, Mansoura University, Egypt, were employed. They were housed for approximately ten days before starting the experiment. The standard conditions for housing are a temperature of 25 ˚C, relative humidity, and a 12/12 h light/dark cycle according to the ‘Guide for the Care and Use of Laboratory Animals’ prepared by the National Academy of Sciences and published by the National Institute of Health (NIH, 1996). Food and water *ad libitum* were permitted.

### Diabetes induction

Diabetes was induced by one intravenous injection of a dose equal to 60 mg/kg of freshly dissolved STZ in ice-cold 0.1 M sodium citrate buffer, pH 4.5^6^. As STZ can induce fatal hypoglycemia because of massive pancreatic insulin release, drinking water was replaced with a 10% glucose solution for the next 24 h to prevent hypoglycemia. Blood glucose levels were determined after 72 h of STZ. Diabetes was evidenced through determination of FBG level from the tail vein using glucose strips and a glucometer (Boehring-Mannheim Diagnostica, Mannheim, Germany). Only rats having a FBG concentration of ≥ 250 mg/dL were considered diabetic and selected to investigate the anti-diabetic effect.

### Experimental protocol

Animals were divided into 5 groups (six per group; one per cage): Diabetes group: diabetic rats received one intravenous injection of a dose equal to 60 mg/kg of freshly dissolved STZ in ice-cold 0.1 M sodium citrate buffer, pH 4.5, Control healthy group: healthy rats administered a corresponding volume of the citrate buffer, Q group: diabetic rats, received one intravenous injection of STZ (60 mg/kg), treated orally via gastric gavage with thymoquinone (freshly suspended in water) at a dose of 30 mg/kg daily for 21 consecutive days, G group: diabetic rats, received one intravenous injection of STZ (60 mg/kg), treated orally via gastric gavage with glycine (freshly dissolved in water) at a dose of 100 mg/kg daily for 21 consecutive days, and Q + G group: diabetic rats, received one intravenous injection of STZ (60 mg/kg), treated orally via gastric gavage with thymoquinone at a dose of 30 mg/kg concomitant with gavage with glycine at a dose of 100 mg/kg daily for 21 consecutive days. Daily food and water intake for each rat was measured through weighing the remaining food in the feeder and measuring the volume of the remaining water in the drinking water bottle, respectively. Animals’ body weight was monitored weekly. At the end of the experiment, rats were fasted for 8 h and then sacrificed by decapitation under anesthesia (ketamine (75 mg/kg) and xylazine (10 mg/kg)). Via the retro-orbital venous plexus, blood was collected; one portion was placed in the commercially available EDTA tubes (1.8 mg of dipotassium salt of EDTA/ml blood), and the other blood portion was allowed to clot at room temperature. Serum was separated for biochemical estimations from the clot by centrifugation at 3000 rpm for 15 min. The liver was collected to make a liver tissue homogenate in ice-cold phosphate-buffered saline.

### Biochemical analyses

FBS, Hb, HbA1C, serum albumin, amylase, TNF-α, IL-10, CK-T, and CK-MB were assessed using kit instructions. Liver homogenate was assessed for oxidative stress (TAC, MDA, and GSH) and glucose metabolism enzymes (glucose-6-phosphate dehydrogenase (G6PD), glucose-6-phosphatase (G6P), and fructose-1,6-bisphosphatase (FBP)) according to kit procedures. HOMA-IR, HOMA %B, HOMA %S, and QUICKI were calculated by the HOMA1 method. The following formulae were used. HOMA-IR = [fasting insulin (µU/L) × fasting glucose (mg/dL)]/405, HOMA %B = [20 × fasting insulin (µU/L)]/[(fasting glucose (mg/dL) × 0.0555) –3.5], and HOMA S% = [1/HOMA-IR] × 100%. Quantitative insulin sensitivity check index (QUICKI) was calculated according to the report by Katz et al.^27^ with the formula QUICKI = 1/(log [fasting insulin in µU/L] + log [fasting glucose in mg/dL]). Insulin resistance was defined as HOMA-IR > 2 as previously reported^28^.

### Histopathology

After decapitation, the pancreas organs were obtained, followed by saline washing, 10% formalin fixation, paraffin wax embedding, microtome sectioning into 5 μm-thick slices, and hematoxylin and eosin (H&E) staining to visualize cellular detail. The examination of these stained pancreas sections was performed in triplicate.

### Statistics

Statistical analysis was conducted using GraphPad Prism version 8.0.2(263) (GraphPad Software, Inc.), utilizing one-way ANOVA followed by Bonferroni post-hoc tests to compare groups. Data were expressed as mean ± standard deviation (SD), with a P-value < 0.05 considered statistically significant.

## Results

### Food intake

**Fig. 1:**
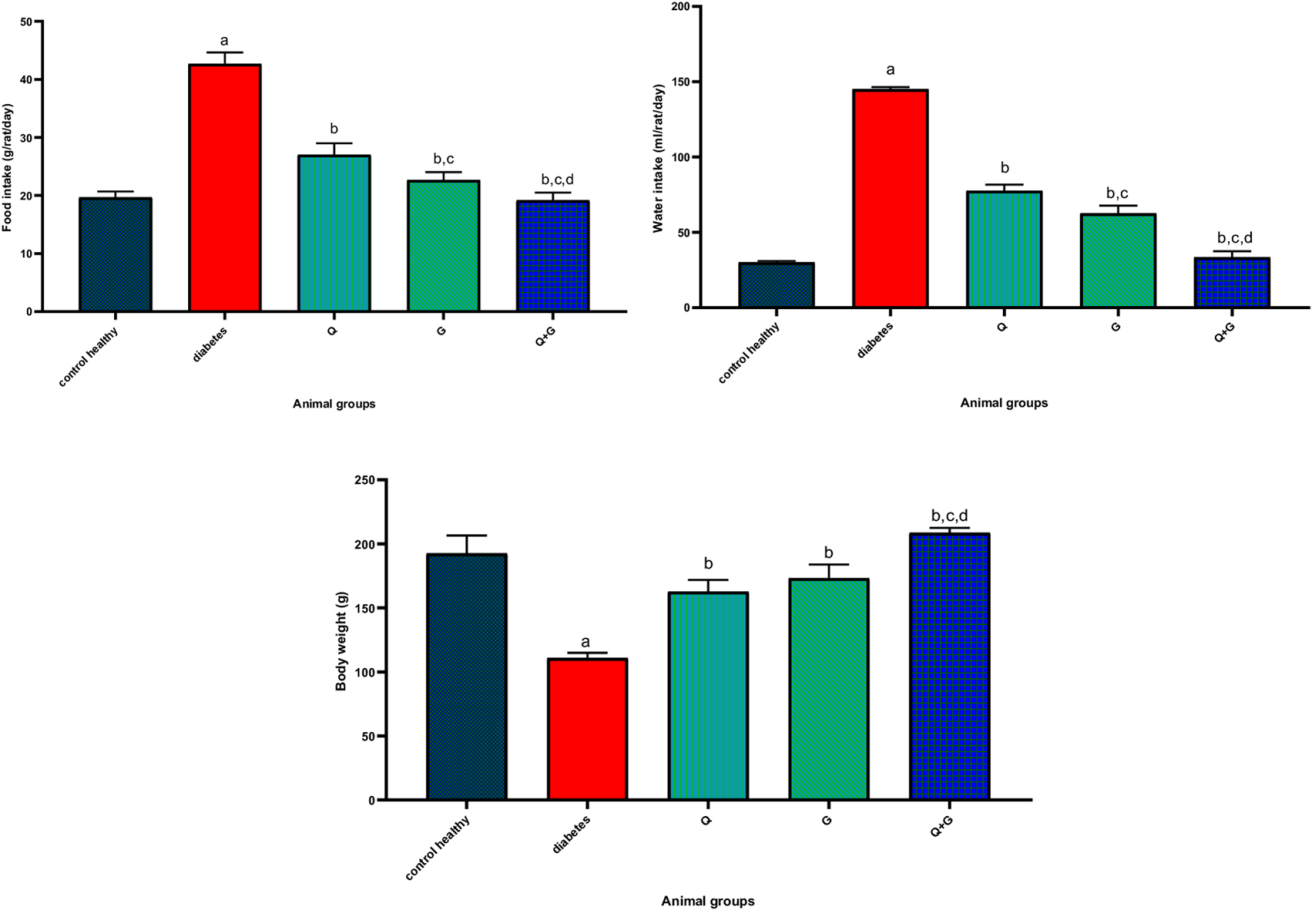
Effect of thymoquinone, glycine, and the combination treatment on food intake, water intake, and body weight after 21 days of treatment. Values represent mean±SD, *n*=6. Differences were assessed using one−way ANOVA test followed by Bonferroni test as a post−hoc test. *P *< 0.05 is significant. Q: thymoquinone; G: glycine. a: there is a significant difference between the group and the control healthy group, b: there is a significant difference between the group and the diabetes group, c: there is a significant difference between the group and the Q group, and d: there is a significant difference between the group and the G group.

As shown in Fig. 1, induction of diabetes significantly increased daily food intake to 42.67 ± 1.97 versus 19.67 ± 1.03 g/rat/day for healthy controls (*P* < 0.05). Thymoquinone, glycine, and the combination lowered it to 27.00 ± 2.00 g/day/rat (*P* < 0.05 vs. diabetes), 22.67 ± 1.367 g/day/rat (*P* < 0.05 vs. diabetes and Q groups), and 19.17 ± 1.02 g/rat/day (*P* < 0.05 vs. diabetes, Q, and G groups).

### Water intake

Figure 1 indicates that diabetic rats consumed significantly more water (145.1 ± 1.36 mL/rat/day) than healthy controls (30 ± 0.95 mL/rat/day, *P* < 0.05). Thymoquinone reduced water intake to 77.5 ± 4.18 mL/rat/day (*P* < 0.05 vs. diabetes), while glycine further lowered it to 62.5 ± 5.24 mL/rat/day (*P* < 0.05 vs. diabetes and Q groups). The combination of thymoquinone and glycine resulted in a consumption of 33.3 ± 4.08 mL/rat/day (*P* < 0.05 vs. diabetes, Q, and G groups).

### Body weight

Diabetes induction caused a significant body weight loss (110.8 ± 4.12 g) versus healthy controls (192.5 ± 14.1 g, *P* < 0.05). Thymoquinone reversed this loss to 162.7 ± 9.14 g (*P* < 0.05 vs. diabetes). Glycine increased the weight to 173.2 ± 10.8 g (*P* < 0.05 vs. diabetes). The Q + G group exhibited the greatest gain (208.7 ± 3.83 g, surpassing both the diabetic and thymoquinone, and glycine groups (*P* < 0.05) and exceeding the healthy control mean (Fig. 1).

### Glucose and HbA1C

**Fig. 2:**
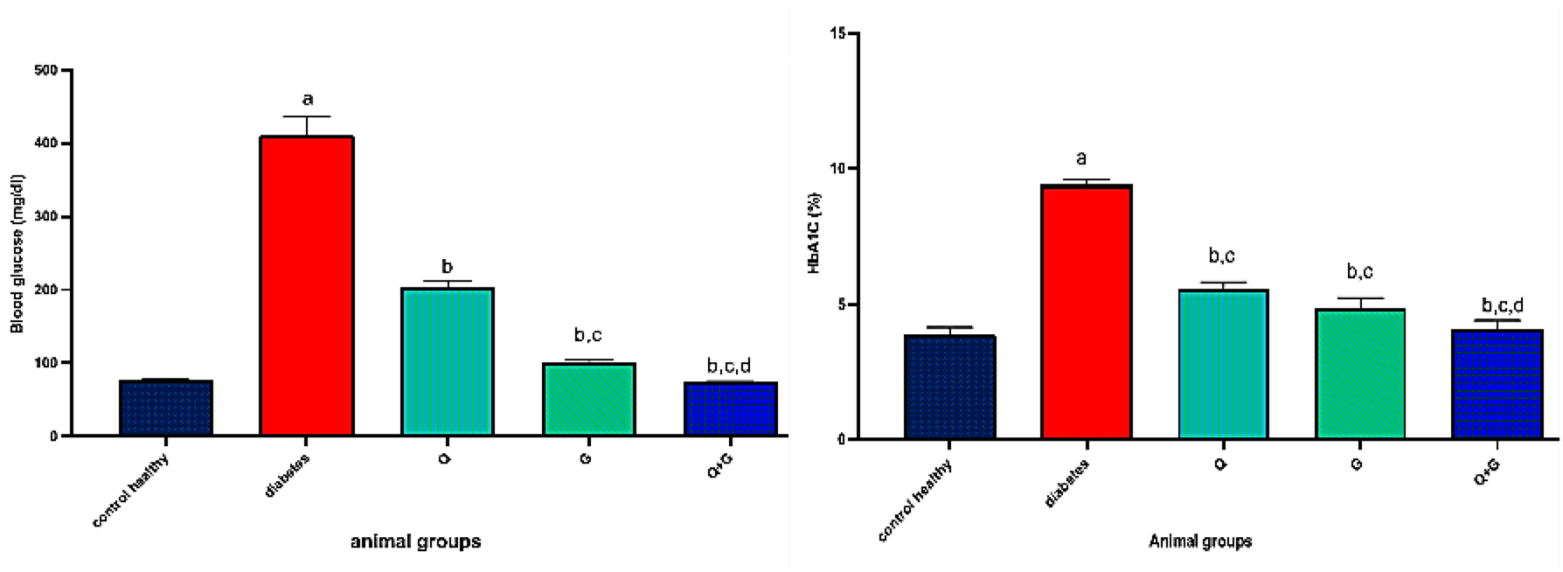
Effect of thymoquinone, glycine, and the combination treatment on blood glucose, Hemoglobin A1C, after 21 days of treatment. Values represent mean±SD, *n*=6. Differences were assessed using one−way ANOVA test followed by Bonferroni test as a post−hoc test. *P *< 0.05 is significant. Q: thymoquinone; G: glycine. a: there is a significant difference between the group and the control healthy group, b: there is a significant difference between the group and the diabetes group, c: there is a significant difference between the group and the Q group, and d: there is a significant difference between the group and the G group.

Diabetic rats showed a significant increase in FBG and HbA1C levels, rising to 410.3 ± 26.39 mg/dL and 9.38 ± 0.23% compared to 2.06 ± 75.67 mg/dL and 3.87 ± 0.27% in healthy rats. When treated with thymoquinone, levels dropped to roughly 204 mg/dL and 5.55% (*P* < 0.05 vs. diabetes). Glycine further lowered levels to 100 ± 4.38 mg/dL and 4.83 ± 0.40% (*P* < 0.05 vs. diabetes and Q groups). The most impressive outcome was seen with the combination, which brought levels down to nearly normal, around 73 mg/dL and 4.07% (*P* < 0.05 vs. diabetes, Q, and G groups, Fig. 2).

### Insulin, HOMA-IR, HOMA %B, HOMA %S and QUICKI

**Fig. 3:**
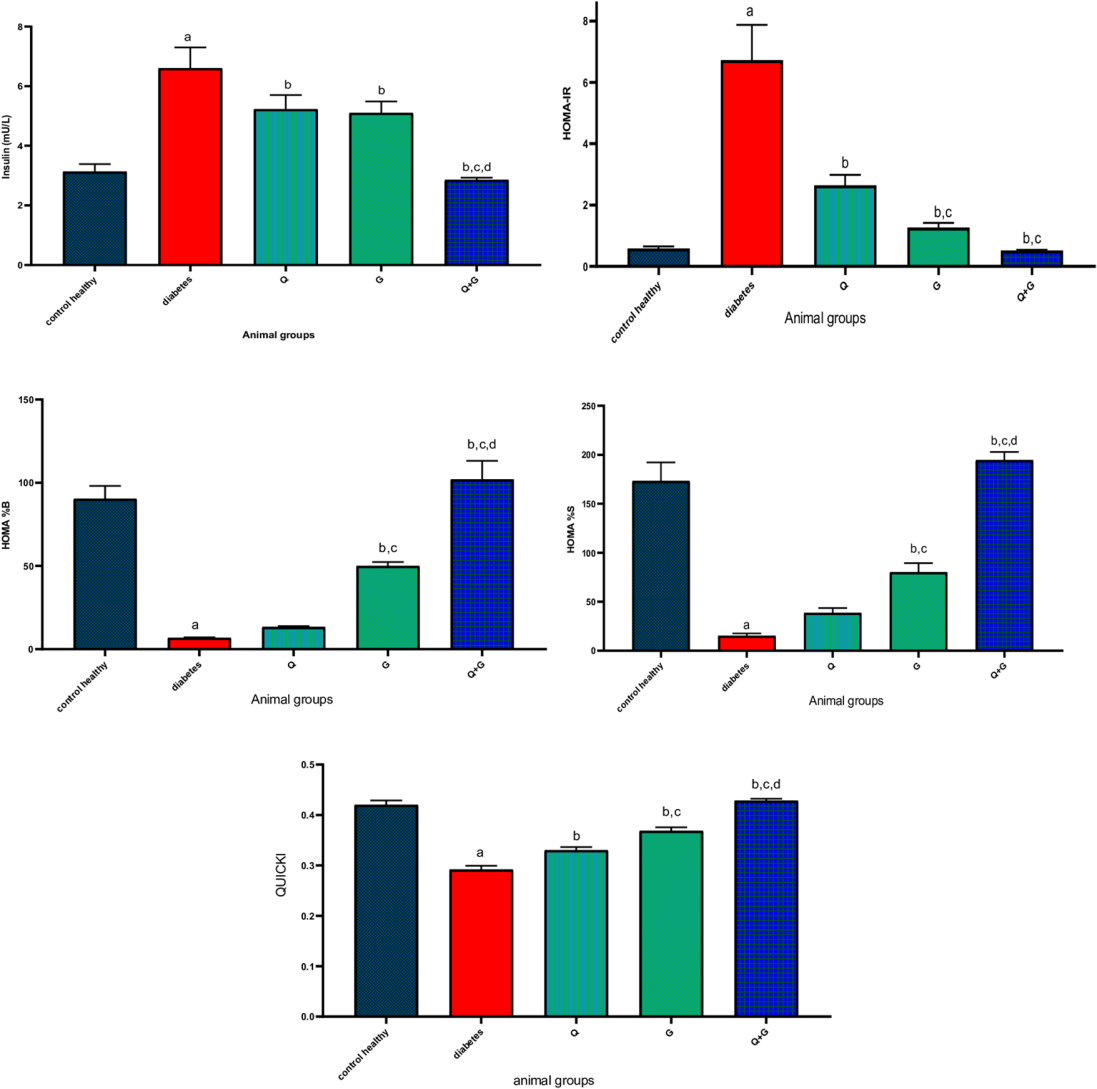
Effect of thymoquinone, glycine, and the combination treatment on serum insulin levels, homeostatic model assessment for insulin resistance (HOMA-IR), HOMA-derived beta-cell function (HOMA %B), HOMA-derived insulin sensitivity (HOMA %S), and quantitative insulin sensitivity check index (QUICKI) after 21 days of treatment. Values represent mean±SD, *n*=6. Differences were assessed using one−way ANOVA test followed by Bonferroni test as a post−hoc test. *P *< 0.05 is significant. Q: thymoquinone; G: glycine. a: there is a significant difference between the group and the control healthy group, b: there is a significant difference between the group and the diabetes group, c: there is a significant difference between the group and the Q group, and d: there is a significant difference between the group and the G group.

As presented in Fig. 3, diabetic rats showed a significant increase in serum insulin levels (6.6 ± 0.7 µg/L) compared to healthy controls (3.13 ± 0.255 µg/L) accompanied by elevated HOMA-IR values (6.725 ± 1.15), impaired β-cell function (HOMA %B: 6.831 ± 0.28), reduced insulin sensitivity (HOMA %S: 15.21 ± 2.4), and low QUICKI score (0.292 ± 0.008). Administration of thymoquinone lowered insulin levels to 5.2 ± 0.48 µg/L and HOMA-IR to 2.64 ± 0.34, with HOMA %S of 38.42 ± 5.09 and QUICKI of 0.33 ± 0.006. Glycine resulted in reductions in insulin levels (5.1 ± 0.39 µg/L), while significantly improving insulin sensitivity (HOMA %S increased to 79.98 ± 9.43%), HOMA-IR values decreased to 1.27 ± 0.15 (*P* < 0.05 vs. diabetic and thymoquinone), and improved β-cell function (HOMA %B: 49.99 ± 2.44%) and QUICKI (0.368 ± 0.008). Importantly, the combination (Q + G) yielded the most substantial improvements across all measured parameters. Insulin levels normalized to 2.85 ± 0.077 µg/L, HOMA-IR decreased to 0.515 ± 0.02, and both insulin sensitivity and β-cell function were markedly restored (HOMA %S: 194.5 ± 8.39; HOMA %B: 101.9 ± 11.31) with a QUICKI of 0.428 ± 0.004 (*P* < 0.05 vs. diabetic, Q, and G).

### Hb, albumin, α-amylase, and CK

As shown in Table 1, Hb levels remained relatively consistent across all groups, with no major deviations. Albumin levels were slightly reduced in diabetic rats (3.023 ± 0.109 g/dL) compared to healthy controls (3.87 ± 0.112 g/dL) (*P* < 0.05). Thymoquinone increased albumin to 3.69 ± 0.16 g/dL (*P* < 0.05 vs. diabetic). Glycine showed similar improvement (3.65 ± 0.1615 g/dL). The Q + G combination achieved the highest albumin level (3.77 ± 0.22 g/dL). α-Amylase levels were significantly decreased in diabetic rats (172.1 ± 24.98 U/L) compared to healthy controls (299.1 ± 7.67 U/L) (*P* < 0.05). Thymoquinone significantly restored α-amylase activity to 264.3 ± 19.72 U/L (*P* < 0.05 vs. diabetic). Glycine further improved levels to 270.1 ± 48.8 U/L. The Q + G combination yielded a value of 279.1 ± 39.12 U/L, closely resembling the healthy baseline.

**Table 1:** Effect on hemoglobin, α-amylase, albumin, and creatine kinase (CK).

Variables	Control healthy	Diabetes	Q	G	Q + G
Hemoglobin (g/dL)	13.43 ± 0.851	14.28 ± 0.570	13.70 ± 0.59	13.87 ± 0.750	14.630 ± 0.49
Albumin (g/dL)	3.870 ± 0.112	3.023 ± 0.109 ^a^	3.690 ± 0.160 ^b^	3.650 ± 0.1615 ^b^	3.770 ± 0.220 ^b^
α-Amylase (U/L)	299.1 ± 7.669	172.1 ± 24.98 ^a^	264.3 ± 19.72 ^b^	270.10 ± 48.80 ^b^	279.1 ± 39.12 ^b^
CK-T (IU/L)	504.3 ± 119.4	2192 ± 308.30 ^a^	749.7 ± 22.49 ^b^	767.40 ± 120.5 ^b^	671.7 ± 58.79 ^b^
CK-MB (IU/L)	885.0 ± 37.15	2003 ± 100.90 ^a^	907.5 ± 58.29 ^b^	947.00 ± 38.51 ^b^	796.7 ± 49.26 ^b, d^

Values represent mean±SD, *n*=6. Differences were assessed using one−way ANOVA test followed by Bonferroni test as a post−hoc test. *P *< 0.05 is significant. Q: thymoquinone; G: glycine.

^a^There is a significant difference between the group and the control healthy group.

^b^There is a significant difference between the group and the diabetes group.

^c^There is a significant difference between the group and the Q group.

^d^There is a significant difference between the group and the G group.

Diabetic rats showed a marked elevation in CK-T levels (2192 ± 308.3 IU/L) compared to healthy controls (504.3 ± 119.4 IU/L) (*P* < 0.05). Treatment with thymoquinone or glycine significantly reduced CK-T to 749.7 ± 22.49 IU/L or 767.4 ± 120.5 IU/L (*P* < 0.05 vs. diabetic). The combination therapy (Q + G) produced a more significant reduction to 671.7 ± 58.79 IU/L (*P* < 0.05 vs. diabetic), nearly matching the control healthy group. CK-MB levels were also significantly increased in diabetic rats (2003 ± 100.9 IU/L) compared to healthy controls (885 ± 37.15 IU/L) (*P* < 0.05). Thymoquinone or glycine reduced it to 907.5 ± 58.29 IU/L or 947 ± 38.51 IU/L (*P* < 0.05 vs. diabetic). The Q + G combination achieved the most substantial improvement, lowering CK-MB to 796.7 ± 49.26 IU/L (*P* < 0.05 vs. diabetic and G), returning values near healthy control levels (Table 1).

### Antioxidants, lipid peroxidation, inflammation, and metabolism enzymes

Diabetic rats exhibited reduced TAC levels (167.2 ± 6.52 nmol/g tissue) compared to healthy controls (234.3 ± 10.27 nmol/g tissue, *P* < 0.05). Treatment with thymoquinone significantly increased TAC (221 ± 27.36 nmol/g tissue, *P* < 0.05 vs. diabetic). Glycine further enhanced TAC levels to 218.3 ± 44.25 nmol/g tissue. The Q + G group showed a TAC value similar to the control group (246.7 ± 25.03 nmol/g tissue). GSH levels were also decreased (2.15 ± 0.25 nmol/g tissue) in the diabetes group compared to controls (5.26 ± 0.82 nmol/g tissue, *P* < 0.05). Thymoquinone moderately increased GSH (2.83 ± 0.34 nmol/g tissue), while glycine showed further increase (6.52 ± 0.75 nmol/g tissue). The Q + G combination produced the most intense enhancement, restoring GSH to 15.54 ± 1.05 nmol/g tissue (*P* < 0.05 vs. all other groups). MDA levels were significantly elevated in the diabetes group (7.72 ± 1.03 nmol/g tissue) compared to healthy controls (3.27 ± 0.50 nmol/g tissue) (*P* < 0.05). All treated groups showed reductions, with Q + G demonstrating the most significant decrease (1.00 ± 0.043 nmol/g tissue), nearly restoring MDA to healthy control levels (*P* < 0.05 vs. diabetic, Q, and G).

Compared to the healthy controls, TNF-α levels were increased while anti-inflammatory IL-10 levels were reduced in diabetic rats (94.17 ± 9.56 ng/dL and 136 ± 20 ng/dL vs. 298.5 ± 46.13 ng/dL and 45.83 ± 6.88 ng/dL, respectively, *P* < 0.05). Thymoquinone and glycine treatments reduced TNF-α and increased IL-10 levels, while the Q + G combination achieved the most improvements; suppressed TNF-α to 139.2 ± 11.14 ng/dL, close to the healthy control level, and boosted protective cytokine IL-10 to 133.8 ± 12.58 ng/dL (*P* < 0.05 vs. diabetic, Q, and G) (Table 2).

**Table 2:** Effect on antioxidants, lipid peroxidation, inflammation, and metabolism enzymes.

Variables	Control healthy	Diabetes	Q	G	Q + G
TAC (nmol/g tissue)	234.3 ± 10.27	167.2 ± 6.520 ^a^	221.0 ± 27.36 ^b^	218.3 ± 44.25 ^b^	246.7 ± 25.03 ^b^
GSH (nmol/g tissue)	5.260 ± 0.820	2.15 0 ± 0.250 ^a^	2.828 ± 0.340	6.523 ± 0.750 ^b, c^	15.54 ± 1.049 ^b, c, d^
MDA (nmol/g tissue)	3.270 ± 0.500	7.720 ± 1.030 ^a^	5.023 ± 0.74 ^b^	1.695 ± 0.320 ^b, c^	1.002 ± 0.043 ^b, c^
TNF-α (ng/dL)	136.0 ± 20.00	298.5 ± 46.13 ^a^	226.2 ± 20.67 ^b^	221.2 ± 18.15 ^b^	139.2 ± 11.14 ^b, c, d^
IL-10 (ng/dL)	94.17 ± 9.560	68.80 ± 45.83 ^a^	91.17 ± 16.02 ^b^	102.0 ± 8.809 ^b, c^	133.8 ± 12.58 ^b, c, d^
G6P (mmol/min/mg protein)	0.1817 ± 0.013	0.420 ± 0.078 ^a^	0.230 ± 0.019 ^b^	0.208 ± 0.0075 ^b^	0.182 ± 0.016 ^b^
FBP (mmol/min/mg protein)	0.325 ± 0.024	0.533 ± 0.025 ^a^	0.337 ± 0.028 ^b^	0.348 ± 0.029 ^b^	0.308 ± 0.004 ^b^
G6PD (mmol/min/mg protein)	509.3 ± 54.95	359.0 ± 16.75 ^a^	488.0 ± 8.695 ^b^	508.5 ± 56.13 ^b^	515.0 ± 19.52 ^b^

Values represent mean±SD, *n*=6. Differences were assessed using one−way ANOVA test followed by Bonferroni test as a post−hoc test. *P *< 0.05 is significant. Q: thymoquinone; G: glycine; TAC: total antioxidant capacity; GSH: glutathione; MDA: malodialdhyde; TNF: tumor necrosis factor; IL: interleukin, G6P: glucose−6−phosphatase; FBP: fructose 1,6−bisphosphatase; G6PD: glucose−6−phosphat dehydrogenase.

^a^There is a significant difference between the group and the control healthy group.

^b^There is a significant difference between the group and the diabetes group.

^c^There is a significant difference between the group and the Q group.

^d^There is a significant difference between the group and the G group.

As presented in Table 2, after diabetes induction, there was a clear change in liver enzyme levels related to glucose metabolism. Significantly, G6P and FBP activities rose, whereas G6PD activity was lowered in diabetic rats (*P* < 0.05 for all vs. control). Treatment with thymoquinone or glycine reduced both G6P and FBP activities while increasing G6PD activity (*P* < 0.05 for all vs. diabetes group). The combination therapy (Q + G) restored G6P activity to 0.1817 ± 0.016 mmol/min/mg protein, FBP activity to 0.308 ± 0.004 mmol/min/mg protein, and G6PD activity to 515 ± 19.52 mmol/min/mg protein, nearly identical to those of the healthy control (*P* < 0.05 for all vs. diabetics).

### Pancreatic histopathological examination

**Fig. 4:**
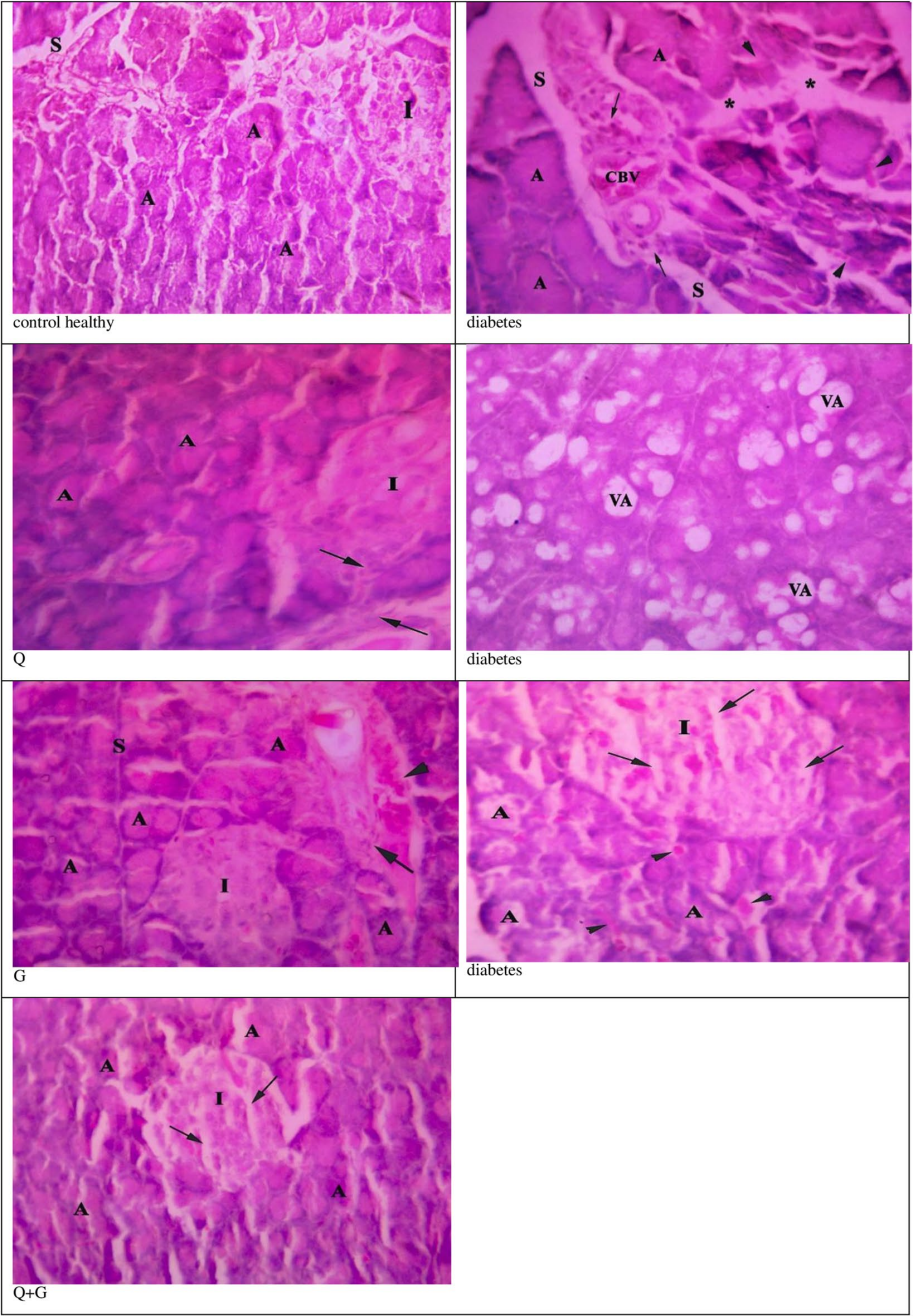
Histopathology photomicrographs of pancreas (H&E, 400X). *Control group* showing normal pancreatic architecture. A: pancreatic acini, S: thin connective tissue septae and I: islet of Langerhans. *Diabetic group* showing deformed pancreatic architecture. A: distorted pancreatic acini, S: widened septae, CBV: congested blood vessel, VA: vacuolated pancreatic acini, star: necrosis of pancreatic acini, Arrow: infiltration of inflammatory cells, Arrow head: hemorrhage, and I: degenerated and widely separated cells in the islets of Langerhans. *Thymoquinone (Q) group* showing improvement with abnormal pancreatic architecture. A: nearly normal acini, I: atrophy of islets of Langerhans, and Arrow: infiltration of inflammatory cells. *Glycine (G) group* showing nearly normal pancreatic architecture with normal A: pancreatic acini I: islets of Langerhans, S: septae. Notice the presence of some inflammatory cells (Arrow) and haemorrhage (arrow head). *Thymoquinone and Glycine (Q+G) group* showing restored and nearly normal pancreatic architecture. A: pancreatic acini, Arrow: degenerated and widely separated cells in the islets of Langerhans (I).

The microscopic examination of the H&E-stained pancreatic sections of the control group showed normal pancreatic tissue histology with normal pancreatic cell aggregation. Despite that, STZ-induced diabetic rats showed significant damage to pancreatic tissues, including distorted pancreatic acini, widened septae, congested blood vessels, necrosis of pancreatic acini, increased infiltration of inflammatory cells, and vacuolated pancreatic acini. In the case of thymoquinone and glycine individual treatment, there was a decrease in the infiltration of inflammatory cells and hemorrhage, and nearly normal acini were observed. Despite this, some symptoms of abnormal pancreatic architecture were still noticed: atrophy of Langerhans islets and widened septae. The combination of thymoquinone and glycine showed better efficient attenuation than the individual treatments (Fig. 4).

## Discussion

The challenge of controlling diabetes is compounded by the high cost and severe side effects of many pharmaceutical drugs, such as hypoglycemia, liver and kidney damage, and even coma, which has spurred research into safer, more effective, and less expensive natural products for antidiabetic use. This work aims to assess thymoquinone and glycine as potential natural alternatives to current treatments. In this study, diabetic rats showed clear symptoms like eating too much, drinking a lot of water, and losing weight. These are all common signs of diabetes, particularly IDDM (type 1), because their bodies can’t use glucose properly. Diabetic rats treated with thymoquinone or glycine, especially in combination, showed improvements in these common diabetes symptoms. Both treatments, particularly the combination, helped the rats reduce their excessive food intake (polyphagia) and the combination helped rats eat back closer to normal. This matches other research saying thymoquinone affects hunger signals like GLP-1^19,29,30^. As for water intake, the diabetic rats drank a lot more to deal with high blood sugar levels, but the different interventions helped reduce excessive water intake (polydipsia). It seems these treatments by lowering blood glucose lead to enhanced relaxation^6^, helped with fluid balance and urination, which is also supported by previous studies on glucose regulation^31^. Weight loss was another big issue for the diabetic rats, but the treated rats experienced gradual weight gain, with the (thymoquinone + glycine) group reaching near-normal weight by the end of the study, indicating improved metabolism and suggesting better insulin sensitivity in the treated rats^14^.

In the present study, the observed metabolic state of diabetic rats reveals hyperglycemia (high blood glucose and HbA1C), insulin resistance (high fasting insulin and HOMA-IR), and impaired β-cell function (low HOMA %B and HOMA %S). Surprisingly, this pattern does not reveal a pure type 1 diabetes model as planned and expected since we used a high dose of STZ (60 mg/kg), but it reveals a hybrid model that mimics both type 1 (impaired β-cell function) and type 2 (insulin resistance) characteristics. High levels of fasting blood glucose and HbA1C indicate a problem with the body’s ability to control blood sugar.

The rise in insulin levels, despite the high dose of STZ (60 mg/kg), may indicate that some residual β-cells have survived^32^ and adapted functionally^33^. This hypothesis suggests that while STZ leads to severe and rapid destruction of β-cells, some surviving, less damaged, or partially resistant β-cells are increasing their insulin production per cell in order to compensate for the massive loss. The combination of low HOMA %B (β-cell function) and low HOMA %S (insulin sensitivity) with high HOMA-IR highlights that the remaining β-cells are under severe stress, failing to maintain homeostasis, which results in hyperglycemia. Elevated insulin alongside hyperglycemia, with insulin resistance implied by high HOMA-IR and low HOMA %B/HOMA %S, indicates the partial compensatory mechanism and a state of insulin resistance rather than simple insulin deficiency. A low QUIKI score, alongside these indicators, contributes to the picture of overall metabolic dysfunction, demonstrating the severity of the condition. Combined treatment with thymoquinone and glycine in the diabetic rats reversed these key diabetes biomarkers, normalizing fasting glucose, reducing HbA1C, minimizing HOMA-IR (insulin resistance), and enhancing HOMA %B (insulin secretion) and HOMA %S (insulin sensitivity) more effectively than either treatment alone. This suggests thymoquinone and glycine work synergistically to improve glycemic control in our diabetic rat model. Thymoquinone helps the body utilize glucose better and reduces oxidative stress^17^. Glycine modulates inflammation and oxidative stress^21^, increases insulin sensitivity, and facilitates glucose uptake^34^. Together, they make a stronger team than either one on its own. This is in agreement with earlier research that demonstrated thymoquinone can improve insulin sensitivity and reduce the liver’s glucose production^13^, consistent with prior studies showing thymoquinone’s protective role against beta-cell apoptosis and its ability to stimulate insulin secretion^35^, and reinforce past studies that reported thymoquinone helps the pancreas function^36^. Again, the observed improvements are likely due to a combination of positive effects. This makes combining natural compounds, such as thymoquinone and glycine, a promising option for helping people manage diabetes in the future.

The current study revealed that thymoquinone, glycine, or a combination showed promise in protecting diabetic rats from various complications by stabilizing hemoglobin levels, improving pancreatic and liver markers (α-amylase and albumin), and reducing cardiac damage by lowering stress enzymes (CK-T and CK-MB). The combination offered more significant and stable improvements in these indicators compared to individual treatments, suggesting their combined antioxidant and anti-inflammatory actions provide complementary protection to the blood, liver, pancreas, and heart from diabetes-related damage^13,31,37–39^. Numerous studies suggest that the strong antioxidant properties and anti-inflammatory effects of thymoquinone and glycine contribute to reduced oxidative stress and enhanced renal tissue health. This may explain the observed improvement in serum albumin levels. By safeguarding the glomerular structure, thymoquinone and glycine help prevent protein loss, thereby maintaining albumin concentrations close to normal levels^17,31,39–41^. Other research reported that glycine may help reduce oxidative damage and restore metabolic balance in liver and heart tissues^42,43^. Al-Fahadi and colleagues (2025) highlighted glycine’s potential in supporting liver health, as it has been associated with improved metabolic balance and reduced cardiovascular risk through its anti-inflammatory and antioxidative roles^42^. On the other side, it was reported that thymoquinone, via activation of the PI3K/Akt pathway in heart cells, supports healthy blood vessels, reduces oxidative stress, suppresses inflammation, and limits cardiomyocyte apoptosis, while also protecting mitochondrial integrity under toxic stress. When administered together, the benefits appear to increase further, likely due to their shared role in reducing stress, enhancing antioxidant defenses, preserving myocardial structure, and reducing cardiac injury markers^44–46^. This indicates thymoquinone plus glycine potential as a protective strategy against diabetes-related complications.

In this study, in line with other research^13,39–41,47,48^, diabetic rats suffered from increased oxidative stress and inflammation, confirmed by increased levels of MDA and TNF-α along with decreased antioxidant capacity (TAC), GSH, and IL-10. Thymoquinone and glycine alone or in combination significantly combat diabetes-induced oxidative stress, lipid peroxidation, and inflammation in diabetic rats. In combination, they exhibited the most significant improvements in antioxidant markers (TAC and glutathione), reduced MDA, and decreased levels of the pro-inflammatory TNF-α while increasing the anti-inflammatory marker IL-10. This synergistic effect suggests that thymoquinone with glycine is a powerful natural approach for managing metabolic stress and immune dysfunction in diabetes.

Another important observation was the increase in G6P levels in diabetic rats, suggesting poor glucose utilization, and the rise in FBP, which promotes glucose production through gluconeogenesis. Conversely, G6PD activity declined significantly in the diabetic rats. G6PD is essential for generating NADPH to battle oxidative stress. Thymoquinone and glycine treatments helped lower both G6P and FBP, and increase G6PD, with the most significant effect observed when used in combination. This indicates improved glycolysis, enhanced glucose uptake, and effective inhibition of excessive hepatic glucose release, thereby helping to control blood sugar levels and providing stronger antioxidant support and better cellular protection. These findings parallel previous reports^49–53^.

In this investigation, the histological study presents great support for our biochemical study. Distorted and vacuolated pancreatic acini, widened septae, congested blood vessels, necrosis of pancreatic acini, and increased infiltration of inflammatory cells signify the remarkable damage in STZ-induced diabetic rats. Individual thymoquinone or glycine treatment resulted in partial improvement; a decrease in inflammatory cell infiltration and hemorrhage, and nearly normal acini; however, pancreatic architecture still shows signs of abnormality. Combined thymoquinone and glycine treatment showed the most improvement, significantly attenuating the damage caused by STZ-induced diabetes.

**Fig. 5:**
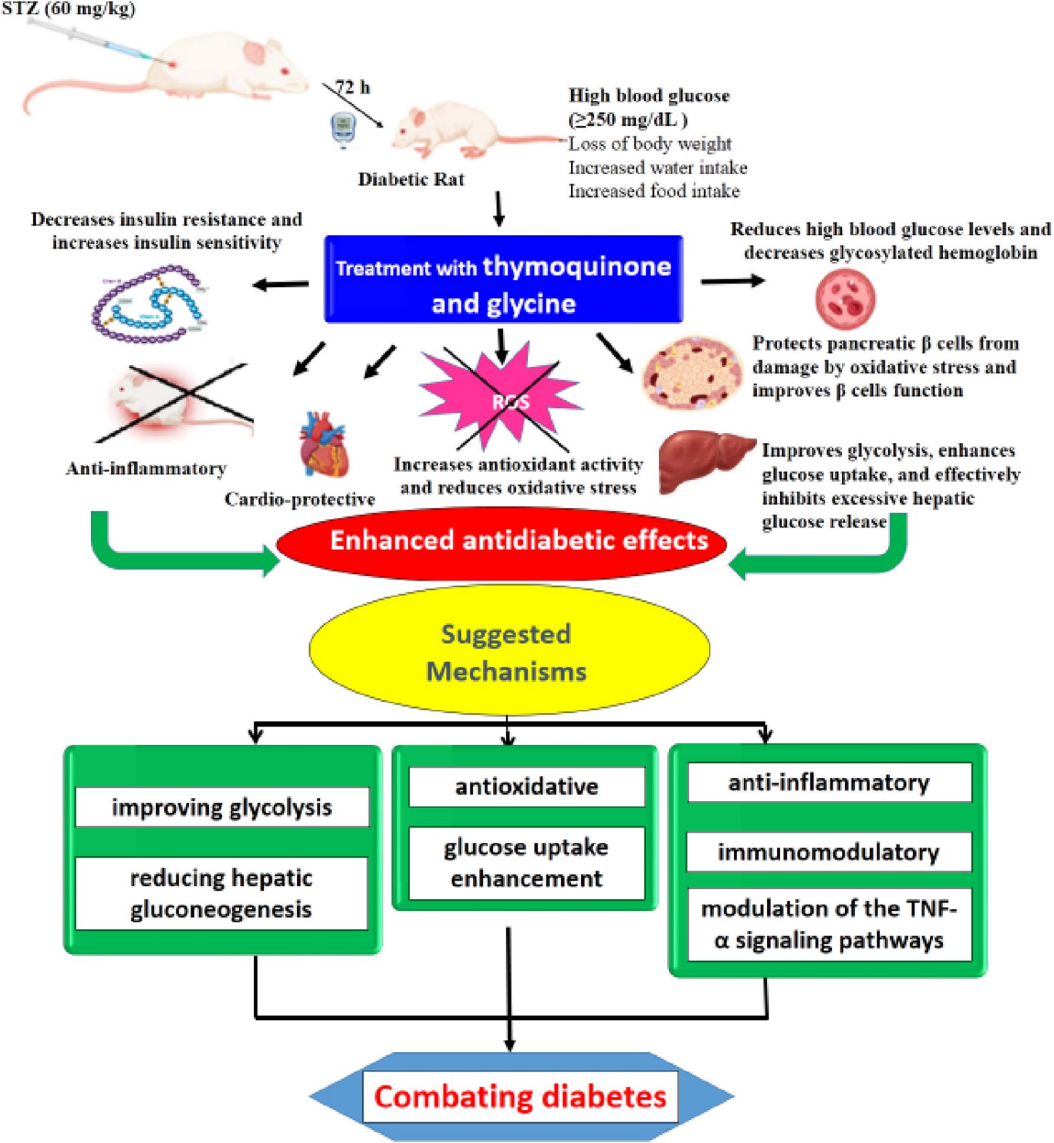
Enhanced antidiabetic effects of thymoquinone and glycine as well as the suggested mechanisms of action.

Overall, this study presents clear evidence that combining thymoquinone with glycine significantly enhances the antidiabetic effects in the STZ-induced diabetes model (Fig. 5). Efficiently, this combination reduced high blood glucose levels, decreased glycosylated hemoglobin, lowered water and food intake, increased insulin sensitivity, improved insulin resistance, protected pancreatic β-cells from damage by oxidative stress and improved β-cells function, alleviated liver and heart damage, improved glycolysis, boosted glucose uptake, and effectively inhibited excessive hepatic glucose release, strengthened the antioxidant defense system, and inhibited inflammation. Depending on these, mechanistically, we can say that it addressed diabetes through multiple pathways, principally decreasing glucose production by the liver (gluconeogenesis), improving the body’s breakdown of glucose (glycolysis), increasing glucose uptake by cells, and modulating the immune response by reducing inflammation, oxidative stress, and the effects of inflammatory signaling pathways that involving TNF-α (Fig. 5).

## Conclusions

In light of our findings regarding the untreated diabetic group: rats showed increased glucose and insulin accompanied by elevated HOMA-IR, impaired β-cell function (HOMA %B: 6.831), reduced insulin sensitivity (HOMA %S: 15.21), and low QUICKI score (0.292), increased TNF-α along with reduced anti-inflammatory IL-10. These results indicate a mixed model combining severe destruction of most β-cells by STZ (necrosis & inflammation, evidenced histopathologically) with insulin resistance rather than solely pure, complete β-cell destruction (type 1) or classical insulin resistance (type 2). This may be temporary until complete β-cell destruction (pure type 1) occurs over time under the effect of the given single STZ dose. This study also demonstrates that thymoquinone and glycine, both individually and in combination, significantly reduce the harmful effects of diabetes in the STZ-induced diabetes rat model. They, especially in combination, effectively ameliorated polyphagia, polydipsia, and weight loss, lowered oxidative stress and inflammation, reduced high glucose and glycosylated hemoglobin levels, enhanced the body’s response to insulin, aided β-cell preservation and improved β-cells function, enhanced the antioxidant defense system, improved glucose metabolism, and protected against lipid peroxidation damage. These positive outcomes are largely due to the potent antioxidant and anti-inflammatory properties of thymoquinone and glycine. This suggests that they may serve as a safe and cost-effective option for managing diabetes in the future.

## Supplementary Information

Below is the link to the electronic supplementary material.

Supplementary Material 1

## Data Availability

Data will be made available upon reasonable request to the corresponding author.
