# Supplementary material for: Combining glycine with thymoquinone offers a promising strategy for diabetes treatment
Source: Sci Rep. 2026 May 19;16:15504. doi: 10.1038/s41598-026-52735-w (PMC13187136; doi:10.1038/s41598-026-52735-w)
Supplement: Supplementary file 1 — Supplementary Material 1 [file 41598_2026_52735_MOESM1_ESM.docx]

**Table 1S** Initial, final, and ∆ weight of the studied groups

| **Variables** | **Control healthy** | **Diabetes** | **Q** | **G** | **Q+G** |
| --- | --- | --- | --- | --- | --- |
| **Initial body weight (g)** | 171.2 ± 6.65 | 154.7 ± 13.5 | 158.3 ± 7.92 | 164.0 ± 7.16 | 172.0 ± 13.77 |
| **Final body weight (g)** | 192.5 ± 14.1 | 110.8 ± 4.12 ^a^ | 162.7 ± 9.136 ^b^ | 173.2 ± 10.8 ^b^ | 208.7 ± 3.83 ^b, c, d^ |
| **∆ weight = (final weight – initial weight)** | +21.3 | -43.9 | +4.4 | +9.2 | +36.7 |

^Values represent mean±SD, n=6. Differences were assessed using one-way-ANOVA test followed by Bonferroni test as a post-hoc test. P<0.05 is significant. Q: thymoquinone; G: glycine.^

^a: there is a significant difference between the group and the control healthy group.^

^b: there is a significant difference between the group and the diabetes group.^

^c: there is a significant difference between the group and the Q group.^

^d: there is a significant difference between the group and the G group.^
